# Observational Arthritis Foundation Internet Study: Physical Activity, Pain, and Physical Function- Study Protocol

**DOI:** 10.1101/2025.08.18.25333813

**Published:** 2025-08-21

**Authors:** Sydney C. Liles, Jennifer Copson, Nurten Gizem Tore, Daniel K. White

**Affiliations:** 1 Department of Physical Therapy, University of Delaware, Newark, DE, USA

**Keywords:** Observational Study, Rheumatic Disease, Physical Activity, Pain, Function

## Abstract

**Background:**

Rheumatic diseases affect approximately 54 million adults in the United States and are a leading cause of pain and disability. Although physical activity is recommended to reduce pain and improve function, rheumatic diseases encompass over 100 conditions with different clinical presentations and medical management, possibly contributing to differences in exercise response. Few studies have included diverse samples across rheumatic diseases making direct comparisons in clinical presentation and activity level difficult.

**Objective:**

To evaluate the feasibility of internet recruitment, validate patient-reported diagnoses, and explore associations of physical activity with pain and function across rheumatic diseases in an online cohort of adults in the United States.

**Methods:**

The Observational Arthritis foundation Internet Study (OASIS): Physical Activity, Pain, and Physical Function study is an online cross-sectional observational study of adults with rheumatic diseases living in the US. Participants will be recruited using Meta ads, Arthritis Foundation mailing lists, and the ResearchMatch database. Participants will be considered eligible if they are ≥ 18 years old, live in the US, and have been diagnosed with a rheumatic disease by a physician. All participants will provide written informed consent and HIPAA release prior to participation. Participants will self-report all rheumatic diseases they have been diagnosed with, and diagnoses will be verified using provider confirmation or electronic medical records. Self-report questionnaires will be used to assess outcomes such as physical activity, pain, physical function, mental and physical health.

**Discussion:**

OASIS is the first study to comparison of physical activity, pain, and physical function across a range of rheumatic diseases in a single cohort. The results from this study could identify differences in presentation or inform tailored exercise recommendations for less common rheumatic diseases.

## Introduction

One in five adults in the United States report being diagnosed with a rheumatic disease,^1,2^ accounting for more than $300 billion in direct medical costs and lost wages in 2013.^3^ Rheumatic diseases, a term which encompasses over 100 conditions including osteoarthritis (OA), rheumatoid arthritis (RA), and lupus, are a leading cause of pain and functional limitations.^1,4–7^ These symptoms interfere with performing daily activities, reduce quality of life, and lead to decreased physical activity.^1,2,8–10^ Reduced physical activity in turn increases risk of other comorbidities such as obesity, cardiovascular disease, and early mortality.^11,12^

Exercise and physical activity are strongly recommended treatments for adults with rheumatic diseases especially OA^13,14^ and RA,^15,16^ two of the most prevalent. Additionally, increased physical activity is associated with reduced risk of mortality, improved muscle strength, and improved psychological health in both the general population^11,12,17,18^ and adults with OA^13,19–21^ and RA.^15,22–25^ Previous research has also shown that increasing physical activity improves pain and function in adults with OA^13,19–21^ and RA.^15,22–25^ Despite these benefits, there is little research examining the effects of physical activity in other types of rheumatic diseases such as lupus, psoriatic arthritis, and gout.

Observational studies are well-suited to study such differences in treatment response and clinical presentation across rheumatic diseases. However, most studies focus on adults with OA or RA, limiting the ability to compare across other rheumatic diseases and to make evidence-based recommendations for less common diseases. Therefore, additional comparisons are needed to understand how physical activity relates to pain and function in adults with different forms of rheumatic disease. This paper outlines the protocol for the Observational Arthritis Foundation Internet Study: Physical Activity, Pain, and Function (OASIS). OASIS aims to evaluate the feasibility of internet recruitment, validate patient-reported diagnoses, and explore associations of physical activity with pain and function across rheumatic diseases.

## Methods

### Study Design

The Observational Arthritis foundation Internet Study (OASIS): Physical Activity, Pain, and Physical Function study is a completely online cross-sectional observational study focusing on adults with different forms of rheumatic disease, such as osteoarthritis (OA), gout, lupus, fibromyalgia, rheumatoid arthritis (RA), and psoriatic arthritis (PsA). This study will measure physical activity, pain, and physical function on adults with rheumatic diseases living in the United States. Study findings will be reported using the STROBE guidelines for observational studies.

### Participants

Individuals will be recruited using Meta advertisements primarily through Facebook, the Arthritis Foundation mailing list, and via the ResearchMatch participant database. ResearchMatch is a national health volunteer registry that was created by several academic institutions and supported by the U.S. National Institutes of Health as part of the Clinical Translational Science Award (CTSA) program. ResearchMatch includes volunteers who have consented to be contacted by researchers about health studies for which they may be eligible. Review and approval for this study and all procedures was obtained from University of Delaware Institutional Review Board. Participants will be included if they are at least 18 years old, live in the United States, and report having a diagnosis of a rheumatic disease provided by a physician. Participants self-report diagnoses of OA, gout, fibromyalgia, RA, psoriatic arthritis (PsA), lupus, or ‘other’ rheumatic disease. Additionally, participants must have internet access, a working email address, feel comfortable answering questions in English, and be willing to have a virtual call to verify their identity. Participants will be excluded if they fail to meet any of the inclusion criteria, have been previously enrolled, or fail the identity verification check.

### Identity Verification Check

We will employ a multi-step strategy to identify individuals who purposefully falsify information to secure payment for participation. We will utilize these strategies to check the validity of reporting from study participants during the enrollment phase. The Internet Protocol (IP) address used by the participant will be analyzed using website-based software to determine if it is located within the United States and does not have evidence of fraud or abuse. Then the informed consent form will be analyzed to ensure the printed name and signed name are consistent and match the name and signature provided on the HIPAA authorization form, if there are inconsistencies in the names, not including addition of middle initials or full versions of first name, the participant will be deemed fraudulent and excluded from the study. Additionally, if the name on the HIPAA form is not the participant’s name and/or the relationship to the participant is something other than self or equivalent (ex., Doctor, friend), then the participant will be excluded from the study. Lastly, we will examine answers to the screening form to ensure that the name provided matches the informed consent and HIPAA forms. If the participant passes all these criteria, they have passed the identity verification check and will be enrolled in the study.

### Enrollment and data collection

Participants who meet study criteria, sign the informed consent and HIPAA authorization forms, and pass the identity verification check will be enrolled. Participant demographics will then be collected, including age, sex, gender, body mass index (BMI), race, whether they were Latino, veteran status, highest level of education, employment status, and annual household income. Self-reported data on their reported rheumatic disease, physical activity, and other outcomes will also be collected.

### Disease Specific Information

Participants will self-report which rheumatic diseases they have been diagnosed with by a physician and the date of diagnosis for each. Participants will select from a list of OA, gout, fibromyalgia, RA, PsA, Lupus, or Other. If other is selected, participants are able to write in any other rheumatic disease they are diagnosed with. Participants will also be able to select multiple diagnoses. If the participant reports being diagnosed with osteoarthritis, they will be asked to report which joints were affected. National Institute for Health and Care Excellence (NICE) criteria for knee OA (≥ 45 years old, activity-related knee pain, and morning stiffness lasting < 30 minutes)^26^ will be assessed in all participants who report OA and specified the knee joint being affected. NICE Criteria have been shown to be sensitive and specific in detecting symptomatic knee OA. Participants will also be asked about previous joint injuries, joint surgeries, and past and current strategies used to manage their disease related symptoms, including options such as physical therapy, diet, corticosteroid injections, immunosuppressants, nonsteroidal anti-inflammatory medications, etc. The use of glucagon-like peptide-1 (GLP-1) agonists will also be assessed.^27,28^

## Primary Outcomes

### Physical Activity

Physical activity will be assessed using the International Physical Activity Questionnaire – Short Form (IPAQ-SF). The IPAQ-SF is a seven-item questionnaire that assesses self-reported physical activity in categories of vigorous activity, moderate activity, walking, and sedentary behavior in the form of sitting.^29–31^ Participants are asked to report the total number of minutes per day and days per week that they engage in vigorous, moderate, and walking activities. The total number of metabolic equivalent units (MET)- minutes in each category is then calculated by multiplying the total number of minutes and days in each category by the MET value for each category (8.0 MET for vigorous, 4.0 for moderate, 3.3 for walking).^32^ Participants are also classified as inactive, minimally active, and health-enhancing physical activity (HEPA) active based on the total combined MET-minutes/week calculations.^32^ HEPA active category is defined as vigorous activity on at least three days and achieving at least 1500 MET-min/wk or 7 or more days of any activity to achieve at least 3000 MET-min/wk.^32^ Minimally active is defined as 3 or more days of vigorous activity for at least 20 min/day, 5 or more days of moderate or walking activity of at least 30 minutes/day, or 5 or more days of any combination of activity achieving at least 600 MET-min/wk.^32^ Inactive is defined as reporting no activity or activity insufficient to meet the criteria for the other two categories.^32^ Sedentary behavior is assessed with one question asking participants to report the total time sitting on an average weekday.^32^ The scoring guidelines for the IPAQ-SF will be utilized for data processing, including handling outliers, truncation of data, and minimal values for activity duration.

### Pain

The primary measure that will be used to assess pain is the Patient-Reported Outcomes Measurement Information System Pain Interference Short Form 8a (PROMIS-PI 8a). The PROMIS-PI is an 8-item questionnaire that assesses self-reported pain interference that has been validated in healthy adults and multiple clinical populations.^33–35^ Additional measures of pain will include the Visual Analog Scale (VAS) for pain and the Michigan Body Map (MBM) for chronic pain. The VAS will be collected from participants who reported a diagnosis of knee OA. This single-item questionnaire asks the participants to make a mark on a 100 mm line to indicate their pain score from 0–100 where zero represents no pain and 100 represents the worst pain imaginable.^36^ The MBM will be collected from all participants, who reported chronic pain, which is pain that is persistent or recurring pain lasting for three months or longer at the time they completed the questionnaire. Participants were asked to select on a human body map all regions where they were experiencing chronic pain. This measure has been validated in adults and is commonly used during the diagnosis of fibromyalgia.^37,38^

### Physical Function

Self-reported physical function will be measured using the Patient-Reported Outcomes Measurement Information System Physical Function Short Form 6b (PROMIS-PF 6b). The PROMIS-PF is a 6-item questionnaire that assesses self-report difficulty performing daily activities such as walking and household chores. This questionnaire has been validated in both healthy and clinical populations.^34,39–41^

## Secondary Outcomes

### Health Status

Overall health status will be measured using the PROMIS Global Health (PROMIS-GH) questionnaire. This 10-item questionnaire assesses overall health and quality of life including subscale scores for mental and physical health. Participants will also complete the Charlson Comorbidity Index (CCI) questionnaire. The CCI is a 17-item questionnaire that creates a weighted score based on a range of comorbidity conditions.

### Kinesiophobia

Kinesiophobia will be assessed using the Tampa Scale of Kinesiophobia (TSK-11). This abbreviated version of the TSK is an 11-item questionnaire that evaluates an individual’s fear-avoidance of movement, fear of work-related activities, and fear of re-injury. This questionnaire has been validated among adults with chronic pain.^42^

### Diagnosis Verification

Self-reported diagnoses will be verified via physician report or electronic medical record (EMR). Participants will specify whether they will provide EMR verification of their diagnoses or if they would prefer the research team to contact their physician for verification. Participants will then either upload a screenshot or scanned document from his/her EMR where their diagnosis is specified, or their physician will complete a provided diagnosis form template. In any instances where the specified diagnosis is unclear, the research team will adjudicate to determine whether the diagnosis can be confirmed.

## Analysis plan

We will use descriptive statistics, mean and standard deviation for continuous variables and frequency and percent for categorical variables, to summarize participant characteristics and study outcomes. To determine feasibility of recruitment, we will calculate the time to recruit 1500 participants and the cost per study completer. We will calculate percent agreement and kappa coefficients with associated 95% confidence interval to determine validity of self-reported diagnoses. We will estimate the cross-sectional association of physical activity, pain, and physical function using linear regression adjusted for potential confounders.

## Data management and Confidentiality

All data will be collected and stored in the REDCap data management system provided by the University of Delaware Center for Human Research Coordination (CHRC). These systems are password protected and require two-factor authentication to access all data.

## Ethics

This observational study received institutional review board approval from the University of Delaware. All participants must provide written informed consent and sign the Health Insurance Portability and Accountability Act (HIPAA) release form before participation. Participants have the right to withdraw at any time without penalty.

## Role of Funding Source

This study is funded by the Arthritis Foundation, which played no role in drafting this manuscript. This study is also supported by the Eunice Kennedy Shriver National Institute of Child Health and Human Development of the National Institutes of Health under award number T32HD007490. The content is solely the responsibility of the authors and does not necessarily represent the official views of the National Institutes of Health.

## Discussion

The OASIS study is a novel observational study of a completely online cohort of adults with rheumatic diseases. This study aims to determine reliability of patient reported diagnoses for rheumatic diseases and address the current gap in literature regarding the relationship of physical activity, pain, and function in adults with less common rheumatic diseases. Previous studies have examined the relationship between physical activity, pain, and function in individuals with OA^13,19–21^ and RA^15,22–25^, however little research on these relationships exists for lupus, psoriatic arthritis, and other rheumatic diseases which limits the ability to provide tailored activity recommendations.

### Strengths

OASIS utilizes a novel completely online recruitment strategy. This allows for recruitment of a geographically diverse sample and inclusion of individuals from rural areas, which are typically understudied. Additionally, this is the first study to include individuals with all rheumatic diseases, allowing for comparisons across rheumatic diagnoses which were not previously possible. The use of validated outcome measures strengthens the reliability of the data, and inclusion of physician verification of diagnoses increases accuracy of rheumatic disease classification.

### Limitations

Since all outcomes will be self-report questionnaires, there is a risk of recall and/or social desirability bias. OASIS is also a cross-sectional study which limits the ability to determine causal relationships between outcomes. Additionally, the online recruitment strategy may risk excluding those less likely to have access to the internet. However, data from the Pew research center shows that in 2024 96% of adults in the US report using the internet.^43^

### Implications

Results from OASIS will inform methods to conduct internet-based recruitment for future studies. Additionally, data could potentially be used to identify differences in clinical presentation and disease specific needs for treatment across rheumatic diseases. Results could also inform tailored exercise recommendations for understudied rheumatic diseases.

## References

[1] Barbour KE, Helmick CG, Boring Michael, Brady TJ. Prevalence of Doctor-Diagnosed Arthritis and Arthritis-Attributable Activity Limitation-United States, 2013–2015. https://www.cdc.gov/nchs/nhis/10.15585/mmwr.mm6609e1 PMC5687192 28278145

[2] RAISING THE GRADE on RHEUMATOLOGY CARE in AMERICA 2022 SEPTEMBER 2022.

[3] Murphy LB, Cisternas MG, Pasta DJ, Helmick CG, Yelin EH. Medical Expenditures and Earnings Losses Among US Adults With Arthritis in 2013. Arthritis Care Res (Hoboken). 2018;70(6):869–876. doi:10.1002/acr.23425 28950426

[4] Fallon EA, Boring MA, Foster AL, Prevalence of Diagnosed Arthritis-United States, 2019–2021.; 2019. https://www.cdc.gov/mmwr/mmwr_continuingEducation.html 10.15585/mmwr.mm7241a1 PMC10578950 37824422

[5] Elgaddal N, Kramarow EA, Weeks JD, Reuben C. Arthritis in Adults Age 18 and Older: United States, 2022 Key Findings Data from National Health Interview Survey.; 2022. https://www.cdc.gov/nchs/products/index.htm.

[6] Yelin E, Murphy L, Cisternas MG, Foreman AJ, Pasta DJ, Helmick CG. Medical care expenditures and earnings losses among persons with arthritis and other rheumatic conditions in 2003, and comparisons with 1997. Arthritis Rheum. 2007;56(5):1397–1407. doi:10.1002/art.22565 17469096 PMC2925686

[7] Williams EM, Walker RJ, Faith T, Egede LE. The impact of arthritis and joint pain on individual healthcare expenditures: Findings from the Medical Expenditure Panel Survey (MEPS), 2011. Arthritis Res Ther. 2017;19(1). doi:10.1186/s13075-017-1230-3 PMC5331686 28245879

[8] Kumthekar A, Pedro S, Michaud K, Physical Activity Habits among Older Adults Living with Rheumatic Disease. Journal of Rheumatology. 2023;50(6):835–841. doi:10.3899/jrheum.211244 36642435

[9] Gabriel SE, Michaud K. Epidemiological studies in incidence, prevalence, mortality, and comorbidity of the rheumatic diseases. Arthritis Res Ther. 2009;11(3). doi:10.1186/ar2669 PMC2714099 19519924

[10] Kłak A, Raciborski F, Samel-Kowalik P. Social implications of rheumatic diseases. Reumatologia. 2016;54(2):73–78. doi:10.5114/reum.2016.60216 27407283 PMC4918047

[11] Piercy KL, Troiano RP, Ballard RM, The physical activity guidelines for Americans. JAMA - Journal of the American Medical Association. 2018;320(19):2020–2028. doi:10.1001/jama.2018.14854 30418471 PMC9582631

[12] Borgundvaag E, Janssen I. Objectively Measured Physical Activity and Mortality Risk Among American Adults. Am J Prev Med. 2017;52(1):e25–e31. doi:10.1016/j.amepre.2016.09.017 27816381

[13] Kolasinski SL, Neogi T, Hochberg MC, 2019 American College of Rheumatology/Arthritis Foundation Guideline for the Management of Osteoarthritis of the Hand, Hip, and Knee. Arthritis and Rheumatology. 2020;72(2):220–233. doi:10.1002/art.41142 31908163 PMC10518852

[14] Bannuru RR, Osani MC, Vaysbrot EE, OARSI guidelines for the non-surgical management of knee, hip, and polyarticular osteoarthritis. Osteoarthritis Cartilage. 2019;27(11):1578–1589. doi:10.1016/j.joca.2019.06.011 31278997

[15] England BR, Smith BJ, Baker NA, 2022 American College of Rheumatology Guideline for Exercise, Rehabilitation, Diet, and Additional Integrative Interventions for Rheumatoid Arthritis. Arthritis Care Res (Hoboken). 2023;75(8):1603–1615. doi:10.1002/acr.25117 37227116

[16] Thoma LM, Li LC, White DK, Oatis C. Physical Therapists Play a Key Role in the Comprehensive Management of Rheumatoid Arthritis. Arthritis Care Res (Hoboken). 2023;75(8):1625–1628. doi:10.1002/acr.25123 37227072

[17] Saint-Maurice PF, Troiano RP, Matthews CE, Kraus WE. Moderate-to-Vigorous Physical Activity and All-Cause Mortality: Do Bouts Matter? J Am Heart Assoc. 2018;7(6). doi:10.1161/JAHA.117.007678 PMC5907548 29567764

[18] Watts EL, Matthews CE, Freeman JR, Association of Leisure Time Physical Activity Types and Risks of All-Cause, Cardiovascular, and Cancer Mortality among Older Adults. JAMA Netw Open. 2022;5(8):E2228510. doi:10.1001/jamanetworkopen.2022.28510 36001316 PMC9403775

[19] White DK, Tudor-Locke C, Felson DT, Walking to meet physical activity guidelines in knee osteoarthritis: Is 10,000 steps enough? Arch Phys Med Rehabil. 2013;94(4):711–717. doi:10.1016/j.apmr.2012.11.038 23228625 PMC3608824

[20] Skou ST, Pedersen BK, Abbott JH, Patterson B, Barton C. Physical activity and exercise therapy benefit more than just symptoms and impairments in people with hip and knee osteoarthritis. Journal of Orthopaedic and Sports Physical Therapy. 2018;48(6):439–447. doi:10.2519/jospt.2018.7877 29669488

[21] Kraus VB, Sprow K, Powell KE, Effects of Physical Activity in Knee and Hip Osteoarthritis: A Systematic Umbrella Review. Med Sci Sports Exerc. 2019;51(6):1324–1339. doi:10.1249/MSS.0000000000001944 31095089 PMC6527143

[22] Katz P, Margaretten M, Gregorich S, Trupin L. Physical Activity to Reduce Fatigue in Rheumatoid Arthritis: A Randomized Controlled Trial. Arthritis Care Res (Hoboken). 2018;70(1):1–10. doi:10.1002/ACR.23230 28378441

[23] Rausch Osthoff AK, Niedermann K, Braun J, 2018 EULAR recommendations for physical activity in people with inflammatory arthritis and osteoarthritis. Ann Rheum Dis. 2018;77(9):1251–1260. doi:10.1136/annrheumdis-2018-213585 29997112

[24] Björk M, Dragioti E, Alexandersson H, Inflammatory Arthritis and the Effect of Physical Activity on Quality of Life and Self-Reported Function: A Systematic Review and Meta-Analysis. Arthritis Care Res (Hoboken). 2022;74(1):31–43. doi:10.1002/acr.24805 34632707

[25] Guglielmo D, Murphy LB, Theis KA, Morbidity and Mortality Weekly Report Walking and Other Common Physical Activities Among Adults with Arthritis-United States, 2019.; 1408. https://cdn-links. 10.15585/mmwr.mm7040a3 PMC8519272 34618794

[26] King L, Stanaitis I, Hung V, VALIDATION OF THE NATIONAL INSTITUTE OF HEALTH AND CARE EXCELLENCE (NICE) CLINICAL CRITERIA FOR THE DIAGNOSIS OF KNEE OSTEOARTHRITIS. Osteoarthritis Cartilage. 2024;32:S212. doi:10.1016/j.joca.2024.02.305

[27] Meurot C, Jacques C, Martin C, Targeting the GLP-1/GLP-1R axis to treat osteoarthritis: A new opportunity? J Orthop Translat. 2022;32:121–129. doi:10.1016/j.jot.2022.02.001 35280931 PMC8888891

[28] Ryan M, Megyeri S, Nuffer W, Trujillo JM. The potential role of GLP-1 receptor agonists in osteoarthritis. Pharmacotherapy. 2025;45(3):177–186. doi:10.1002/phar.70005 39980227

[29] Smith RD, Dziedzic KS, Quicke JG, McHugh GA, Healey EL. Comparing of reliability, construct validity and responsiveness of the IPAQ-SF and pase in adults with osteoarthritis. Osteoarthritis Cartilage. 2020;28:S428. doi:10.1016/j.joca.2020.02.663 33683799

[30] Joseph KL, Dagfinrud H, Christie A, Hagen KB, Tveter AT. Criterion validity of The International Physical Activity Questionnaire-Short Form (IPAQ-SF) for use in clinical practice in patients with osteoarthritis. BMC Musculoskelet Disord. 2021;22(1). doi:10.1186/s12891-021-04069-z PMC7916302 33639913

[31] Lee PH, Macfarlane DJ, Lam TH, Stewart SM. Validity of the international physical activity questionnaire short form (IPAQ-SF): A systematic review. International Journal of Behavioral Nutrition and Physical Activity. 2011;8. doi:10.1186/1479-5868-8-115 PMC3214824 22018588

[32] Guidelines for Data Processing and Analysis of the International Physical Activity Questionnaire (IPAQ)-Short Form.; 2004. www.ipaq.ki.se.

[33] Askew RL, Cook KF, Revicki DA, Cella D, Amtmann D. Evidence from diverse clinical populations supported clinical validity of PROMIS pain interference and pain behavior. J Clin Epidemiol. 2016;73:103–111. doi:10.1016/j.jclinepi.2015.08.035 26931296 PMC4957699

[34] DiRenzo D, Saygin D, de Groot I, Reliability and validity of PROMIS physical function, pain interference, and fatigue as patient reported outcome measures in adult idiopathic inflammatory myopathies: International study from the OMERACT myositis working group. Semin Arthritis Rheum. 2023;58. doi:10.1016/j.semarthrit.2022.152111 PMC10354786 36410180

[35] Crins MHP, Terwee CB, Westhovens R, First Validation of the Full PROMIS Pain Interference and Pain Behavior Item Banks in Patients With Rheumatoid Arthritis. Arthritis Care Res (Hoboken). 2020;72(11):1550–1559. doi:10.1002/acr.24077 31562795

[36] Delgado DA, Lambert BS, Boutris N, Validation of Digital Visual Analog Scale Pain Scoring With a Traditional Paper-based Visual Analog Scale in Adults. J Am Acad Orthop Surg Glob Res Rev. 2018;2(3). doi:10.5435/JAAOSGlobal-D-17-00088 PMC6132313 30211382

[37] Brummett CM, Bakshi RR, Goesling J, Preliminary validation of the Michigan Body Map. Pain. 2016;157(6):1205–1212. doi:10.1097/j.pain.0000000000000506 26835782 PMC4868633

[38] Brummett C, Clauw D, Williams D, Goesling J, Hassett A. Self-Report of multiple body regions of pain using the Michigan Body Map is associated with worse pain and suffering. J Pain. 2012;13(4):S28. doi:10.1016/j.jpain.2012.01.119

[39] Voshaar MAHO, ten Klooster PM, Glas CAW, Validity and measurement precision of the PROMIS physical function item bank and a content validity-driven 20-item short form in rheumatoid arthritis compared with traditional measures. Rheumatology (United Kingdom). 2015;54(12):2221–2229. doi:10.1093/rheumatology/kev265 26224306

[40] Jensen RE, Potosky AL, Reeve BB, Validation of the PROMIS physical function measures in a diverse US population-based cohort of cancer patients. Quality of Life Research. 2015;24(10):2333–2344. doi:10.1007/s11136-015-0992-9 25935353 PMC5079641

[41] Wahl E, Gross A, Chernitskiy V, Validity and Responsiveness of a 10-Item Patient-Reported Measure of Physical Function in a Rheumatoid Arthritis Clinic Population. Arthritis Care Res (Hoboken). 2017;69(3):338–346. doi:10.1002/acr.22956 27332620 PMC6369925

[42] Tkachuk GA, Harris CA. Psychometric properties of the tampa scale for kinesiophobia-11 (TSK-11). Journal of Pain. 2012;13(10):970–977. doi:10.1016/j.jpain.2012.07.001 23031396

[43] Pew Research Center. Internet, Broadband Fact Sheet.; 2024. Accessed July 31, 2025. https://www.pewresearch.org/internet/fact-sheet/internet-broadband/

